# Whey protein isolate enhances the growth and probiotic features of *Lacticaseibacillus rhamnosus* GG

**DOI:** 10.1007/s00253-026-13945-4

**Published:** 2026-07-09

**Authors:** Adrienne B. Narrowe, LinShu Liu, Venkateswari J. Chetty, Karley K. Mahalak, Jenni Firrman, Johanna M. S. Lemons

**Affiliations:** 1 Dairy and Functional Foods Research Unit, Eastern Regional Research Center, Agricultural Research Service, United States Department of Agriculture, Wyndmoor, PA USA; 2 Foreign Animal Disease Research Unit, National Bio and Agro-Defense Facility, Agricultural Research Service, United States Department of Agriculture, Manhattan, KS USA

**Keywords:** *Lacticaseibacillus rhamnosus*, Probiotic, Whey protein, P40, Pilus

## Abstract

**Abstract:**

*Lacticaseibacillus rhamnosus* strain GG (LGG) is a broadly used probiotic with several unique features that help it provide beneficial effects to its host. Key among the probiotic features of LGG is the production of bioactive metabolites and secreted proteins such as p40 and p75. The ability of LGG to persist in the gastrointestinal tract depends primarily on its ability to adhere to the gut mucosa via the generation of adhesion pili. While LGG is already used as a probiotic, potential still exists for optimization of the metabolic state of LGG to further enhance its probiotic capacity. Here, we evaluated the ability of whey protein isolate to enhance the cell growth and probiotic effects of LGG. RNA sequencing and untargeted metabolomics revealed that WPI supplementation enhanced LGG growth and metabolic activity and increased the transcription of genes for the production of adhesion pili, key secreted proteins, and beneficial metabolites. These results indicate that whey protein is a viable supplement option for use with LGG and may help to boost the probiotic activity and growth of LGG within the gastrointestinal tract.

**Key points:**

• *Whey protein isolate supplementation increases Lacticaseibacillus rhamnosus GG growth.*

• *Transcription of genes for probiotic features is amplified by the addition of WPI.*

• *Transcriptomics and metabolomics suggest the protein produces the beneficial effects.*

**Supplementary Information:**

The online version contains supplementary material available at https://doi.org/10.1007/s00253-026-13945-4.

## Introduction

Lactobacilli are important for their ability to ferment a variety of carbon sources to produce lactic acid and other metabolic end products. These bacteria are found in multiple habitats including mammalian guts and dairy ecosystems and exhibit different genomic adaptations depending on the environment in which they live (Douillard et al. 2013; Sun et al. 2015, Pasolli, De Filippis et al. 2020). Lactic acid bacteria are commercially valuable as starter bacteria in the dairy industry and are also well known for their health benefits as probiotics. There is much variability among lactic acid bacteria, including within the *Lactobacillaceae* family, but until recently many of these lactic acid bacteria were classified together into the genus *Lactobacillus*. It has since been found that this genus encompassed more genomic and phylogenetic diversity than a typical bacterial family, leading to it being reclassified (Sun et al. 2015). A strain of one of these renamed species, *Lacticaseibacillus rhamnosus* GG (LGG), is among the best known and broadly used probiotic organisms (Doron et al. 2005; Segers and Lebeer 2014; Capurso 2019). Beneficial effects of LGG have been reported to include the inhibition of pathogens, the modulation of the host immune response via the production of bioactive metabolites, and the enhanced growth and protection of the gut epithelium (Silva et al. 1987; Yan and Polk 2002, 2012; Yan et al. 2013; Zhou et al. 2022; Olvera-Rosales et al. 2023; Suntornsaratoon et al. 2024), and even enhanced bone growth (Wei et al. 2023). The beneficial effects of probiotics can depend on their growth phase and metabolic state. Thus, enhancing the metabolic condition of the probiotic with a subsequent benefit to the host by using prebiotic or other types of supplementation is a desired goal (Gibson et al. 2017; Fenster et al. 2019; Lakshmi Ragavan and Das 2021). Expanding upon this, here we explore the effects of commercial whey protein isolate (WPI) on the growth and metabolism of LGG as a potential substrate to enhance its effectiveness.

Whey is the residual liquid from cheese production. Whey protein isolate is further processed from whey and can be used as a dietary protein source (Rocha-Mendoza et al. 2021). It has had most of the fats and lactose removed and should be > 90% protein by weight, with beta-lactoglobulin comprising the most abundant protein type (Madureira et al. 2007). Whey protein has been previously found to exhibit prebiotic potential for many of the inhabitants of the gastrointestinal tract (Sanchez-Moya et al. 2017). Lactobacilli are auxotrophic for many amino acids and consequently have extensive proteolytic systems, though the specificity of these systems and their relative activity can vary greatly by species or strain (Solieri et al. 2015). Prior research involving lactobacilli and whey protein has examined the ability of lactobacilli to hydrolyze whey proteins to produce bioactive peptides, as well as the ability of lactobacilli to reduce the antigenicity of the whey protein lactoglobulin (Raveschot et al. 2018; Olvera-Rosales et al. 2023). Research has been performed describing a time course of growth of LGG in whey solution, but not in comparison to untreated controls (Laakso et al. 2011). Whey protein has also been explored as a potential encapsulation agent for the delivery of probiotic LGG (Yin et al. 2024), but not as an additional nutrient source during the delivery of this probiotic strain.

In this experiment, we sought to evaluate the ability of WPI to enhance LGG’s growth and beneficial effects, focusing on its ability to enhance LGG adhesion, and induce the probiotic effects of LGG. To test this, cultures of LGG were grown anaerobically for 31 h in de Man, Rogosa, and Sharpe (MRS) as a control and in MRS broth supplemented with 1% WPI. The effects of WPI on LGG growth were measured using cell counts via flow cytometry, and the effects of WPI on LGG metabolism were assessed using RNA sequencing in conjunction with untargeted metabolomics analysis.

## Methods and materials

### LGG growth in MRS and WPI

Overnight cultures from frozen stock of a laboratory strain of *Lacticaseibacillus rhamnosus* GG were grown anaerobically in de Man, Rogosa, and Sharpe broth (MRS) (BD, Franklin Lakes, NJ, USA). Prior to use, the MRS broth and MRS agar were autoclaved at 121 °C, 12 psi for 30 min and then degassed with N2 gas for an hour at 63 °C. To get a single colony, a loopful of the overnight grown culture was streaked on MRS agar plates and incubated anaerobically for 32 h. A single colony was cultured anaerobically in MRS broth for 24 h and sequential subculturing was performed two times. A fraction of the subculture (500 µL) was inoculated into 100 mL of MRS broth in a Hungate bottle and incubated at 37 °C, 225 rpm in an anaerobic incubator for 12 h. A fraction of the culture was used to determine the density (McFarland’s units) using a densitometer (DEN-1; Thomas Scientific, Swedesboro, NJ, USA). The culture (45 mL) was dispensed into two 50-mL sterile conical tubes under anaerobic conditions and then centrifuged for 10 min at 4 °C at 5000 rpm. The pellet was resuspended in 25 mL of MRS broth, and this process was repeated in 25 mL of MRS broth, and the density was measured again. The culture was diluted 1:3 ratio with MRS broth, vortexed, and density was measured (concentration of 2.0 × 10^7^ cells/mL). This was used as LGG stock and 0.25 mL was used as inoculum in the experiment to have the required concentration of ~1.0 × 10^6^ cells/mL.

The experiment was conducted in triplicates with a 5.0-mL culture volume and the samples were taken at 0-, 8-, 12-, 16-, and 31-h timepoints. The LGG growth media for the experiment was composed of 15 Hungate tubes with 3.75 mL MRS broth and 1.0 mL PBS, and 0.25 ml LGG stock (control); 12 Hungate tubes with 3.75 mL MRS broth, 1.0 mL whey protein isolate (WPI) (NOW Foods, Bloomingdale, IL, USA) (5% w/v) in PBS, and 0.25 mL LGG stock (WPI). The stock solution of WPI was made in sterile phosphate buffered saline, PBS (1X, NaCl (137 mm); KCl (2.7 mM); Na2HPO4 (10 mM); and KH2PO4 (1.8 mM), pH 7.2). Additive sterility was inferred by triplicate control incubations of WPI solution or SPI solution in MRS broth which indicated no changes in optical density at 12 and 16 h. The tubes were incubated anaerobically at 37 °C without shaking for 31 h in the Bactron anaerobic chamber [Supplementary Fig. 1]. Cultures of LGG supplemented with soy protein isolate (SPI) (NOW Foods, Bloomingdale, IL, USA) were prepared in the same manner and grown and analyzed concurrently with the WPI experiment.

### Flow cytometry

The LGG growth curves were established by counting the cells at 0-, 8-, 12-, 16-, and 31-h timepoints using BD FACS Melody (BD, Franklin Lakes, NJ, USA). From each experimental sample, 1.0 mL of the culture was pelleted down at 10,000g for 10 min at room temperature. The cell pellet was resuspended in 1.0-mL sterile 0.85% sodium chloride solution. Serial dilution was performed with 0.85% sodium chloride solution, and 1.0 mL of the diluted culture was used for each assay. To each tube, 1.0 µL of SYTO® BC bacterial stain (Component A, Bacteria Counting kit, B7277, Invitrogen) was added followed by incubation at 37 °C for 5 min in dark. Next, 10 µL of microsphere suspension (Component B, Bacteria Counting kit, B7277, Invitrogen) was added and mixed well and analyzed by flow cytometry using BD FACS Melody.

### Reference genome sequencing and assembly

To verify the identity and the continued presence of genes of interest, whole-genome sequencing of the *Lacticaseibacillus rhamnosus* GG isolate used in this experiment was performed and the genome assembled for use as the reference in the RNA-Seq experiment. Total genomic DNA was extracted and sequenced by SeqCenter (Pittsburgh, PA, USA) using the Illumina DNA Prep kit (Illumina, Inc., San Diego, CA, USA) and 2 × 151 bp paired-end sequencing was performed on the Illumina NextSeq 2000 (Illumina, Inc., San Diego, CA, USA). The resulting DNA sequencing data was preprocessed using BBDuk v.38.79 (https://sourceforge.net/projects/bbmap) to quality trim reads and remove adapters. A reference-guided assembly was generated using SPADES v. 3.15.4 (Prjibelski et al. 2020) with the *Lacticaseibacillus rhamnosus GG* ATCC 53103 genome used as the reference (ATCC, Gaithersburg, MD, USA). Gene prediction and annotation was performed using Prokka v.1.14.5 (Seemann 2014), with genes from this assembly assigned the prefix “LRGG,” and these called genes were mapped to the reference LGG genome ATCC 53013 genes using both amino acid and nucleotide BLAST v.2.15.0 (Altschul et al. 1990; Camacho et al. 2009). The mapping of the genes from this experiment to the reference genes of ATCC 53103 (ATCC_53103/GCA_000026505.1) and to LHRM (DDBJ AP011548/GCA_000011045.1) is found in File S2.

### RNA sequencing and analysis

RNA extraction, library preparation, and sequencing were performed by Azenta Life Sciences (South Plainfield, NJ, USA) according to their standard protocols. Briefly, RNA was extracted from samples stored in RNA Later using the QIAGEN RNeasy minikit (Qiagen, Hilden, Germany). Samples were quantified using the Qubit 2.0 Fluorometer (Thermo Fisher Scientific, Waltham, MA, USA) and checked using the 4200 TapeStation (Agilent Technologies, Palo Alto, CA, USA). DNA contaminants were removed using Thermo Fisher Scientific TURBO DNase. rRNA depletion was performed using the QIAGEN FastSelect Bacteria Kit after which sequencing library preparation was performed using the NEBNext Ultra II RNA Library Preparation Kit for Illumina (NEB, Ipswich, MA, USA). Sequencing libraries were validated and quantified as above and additionally using qPCR (KAPA Biosystems, Wilmington, MA, USA). Samples were multiplexed and sequenced on an Illumina NovaSeq to generate 2 × 150 PE reads and were demultiplexed to per-sample fastq files using the Illumina bcl2fastq program (v. 2.20).

Raw sequencing data was preprocessed using BBDuk v.38.79. Kallisto v. 0.48.0 (Bray et al. 2016) was used to quantify reads vs. the assembled reference genome. DESeq2 v. 1.44.0 (Love et al. 2014) was used to test for significantly different levels of transcription in binary comparison between timepoints within treatment types or by treatment type within timepoints using Wald test of log2 fold change with Benjamini Hochberg false discovery rate correction. Variance stabilized counts were extracted using DESeq2 for use in visualizations.

### Untargeted metabolomics

Untargeted metabolomics analysis was performed by Metabolon, Inc. (Morrisville, NC, USA) according to company protocols. Briefly, samples were extracted and prepared using the MicroLab STAR® system (Hamilton Company, Reno, NV, USA) for analysis using two separate reverse phase (RP)/UPLC-MS/MS methods with positive ion mode electrospray ionization (ESI), for analysis by RP/UPLC-MS/MS with negative ion mode ESI, and for analysis by HILIC/UPLC-MS/MS with negative ion mode ESI. UPLC-MS/MS was performed using Waters ACQUITY (Milford, MA, USA) ultra-performance liquid chromatography (UPLC) and a Q-Exactive high resolution/accurate mass spectrometer (Thermo Fisher Scientific, Waltham, MA, USA) interfaced with a heated electrospray ionization (HESI-II) source and Orbitrap mass analyzer operated at 35,000 mass resolution. The dried sample extracts were then reconstituted in solvents compatible with each of the four methods. Following reconstitution in appropriate solvents, aliquots were analyzed as follows: one aliquot was analyzed using acidic positive ion conditions, chromatographically optimized for more hydrophilic compounds. In this method, the extract was gradient eluted from a C18 column (Waters UPLC BEH C18-2.1 × 100 mm, 1.7 µm) using water and methanol, containing 0.05% perfluoropentanoic acid (PFPA) and 0.1% formic acid (FA). Another aliquot was also analyzed using acidic positive ion conditions, chromatographically optimized for more hydrophobic compounds wherein the extract was gradient eluted from the same C18 column using methanol, acetonitrile, water, 0.05% PFPA, and 0.01% FA and was operated at an overall higher organic content. Another aliquot was analyzed using basic negative optimized conditions using a separate dedicated C18 column. The basic extracts were gradient eluted from the column using methanol and water, however with 6.5 mM ammonium bicarbonate at pH 8. The fourth aliquot was analyzed via negative ionization following elution from a HILIC column (Waters UPLC BEH Amide 2.1 × 150 mm, 1.7 µm) using a gradient consisting of water and acetonitrile with 10 mM ammonium formate, pH 10.8. Raw data extraction, peak identification, QC, and compound annotation were conducted using Metabolon’s in-house software and spectral libraries.

### Visualizations and other statistical methods

Plots were generated using RStudio v 2024.09.0, build 375 (R version 4.4.1) (R-Core-Team 2021) using packages: tidyverse (Wickham 2019), pheatmap (Kolde 2019), and cowplot (Wilke 2024).

## Results

### Effects of WPI on LGG growth

**Fig. 1:**
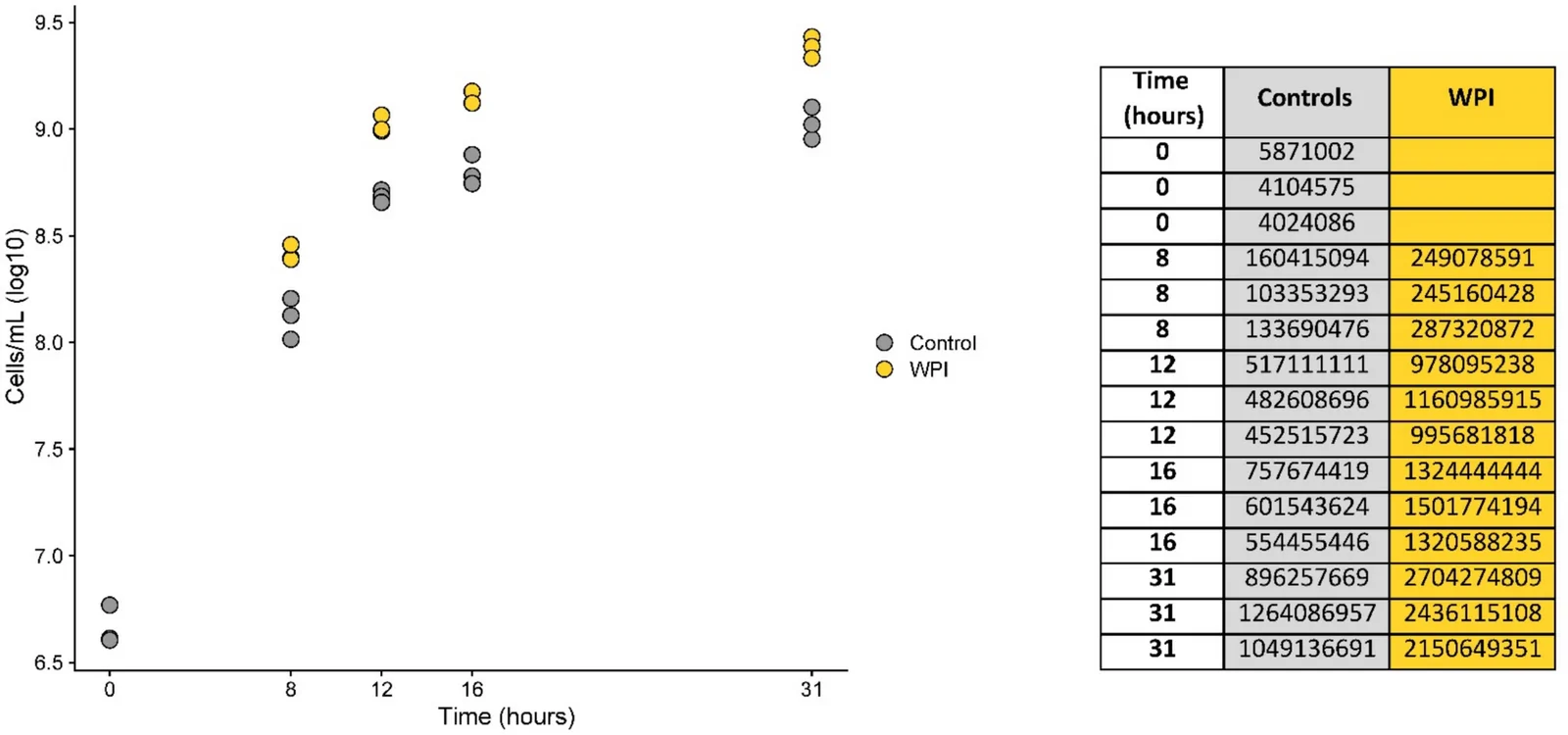
Flow cytometry–based growth curve of LGG in MRS broth (control) and MRS broth supplemented with whey protein isolate (1% WPI) over 31 h of growth. Counts are shown in the table at right

Supplementation with WPI enhanced LGG growth. Within 8 h of incubation with WPI, the treated cultures were significantly higher in cell numbers as measured using flow cytometry (2.61 × 10^8^ ± 1.9 × 10^7^, *p*-value = 0.02) than the unamended samples (1.33 × 10^8^ ± 2.33 × 10^7^) and this trend continued over the course of the experiment with cell counts in WPI-amended samples more than double those in the control samples at the final timepoint (2.43 × 10^9^ ± 2.26 × 10^8^ vs. 1.07 × 10^9^ ± 1.51 × 10^8^, adjusted *p*-value < 0.01 for all remaining timepoints) (Fig. 1, File S1). Beyond the large initial increase in cell counts from 0 to 8 h, significant increases occurred within both treatment groups from 8 to 12 h, and from 16 to 31 h; however, only the WPI samples increased significantly from 12 to 16 h. While larger in cell numbers, the WPI growth curve tracked the overall growth curve of the control samples (Pearson *R* = 0.98, *p* = 3.3 × 10^–8^), with an estimated doubling time of 1.38 h for the WPI-treated samples vs. 1.66 h in the control samples. This suggests that the addition of WPI may have provided an additional nutrient source beyond the MRS which corresponded to higher overall cell counts observable by 8 h after its addition and an initially increased growth rate. A concurrent experiment using soy protein isolate resulted in higher cell counts than controls, but lower counts than in the WPI-treated samples [Supplementary Fig. 2].

### Transcriptional changes with WPI addition

**Fig. 2:**
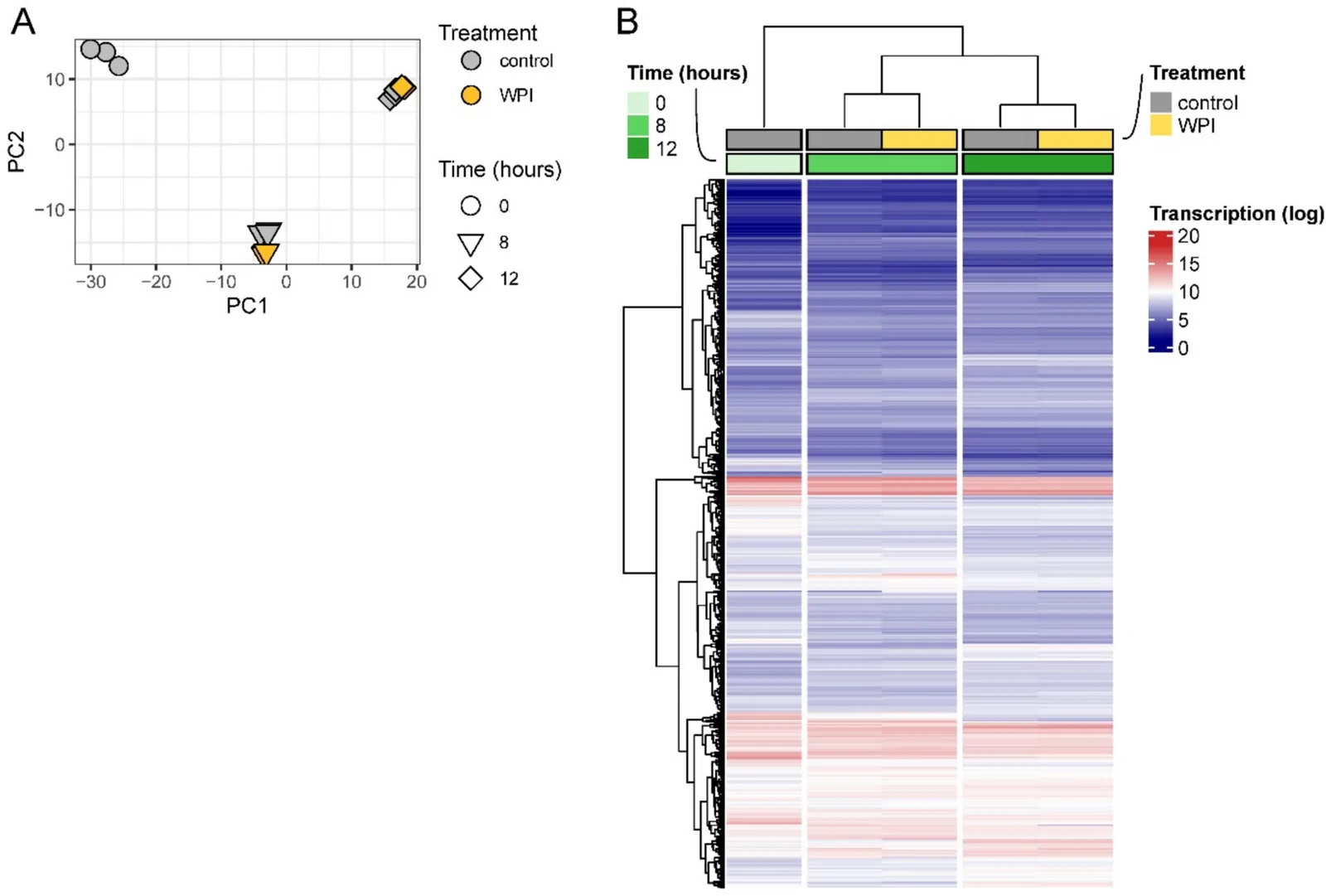
**A** PCA of RNA-Seq samples representing 0, 8, and 12 h of growth in control and WPI-treated samples. Samples cluster by timepoint more than treatment. **B** Heatmap showing all transcribed genes hierarchically clustered by samples and by gene

**Fig. 3:**
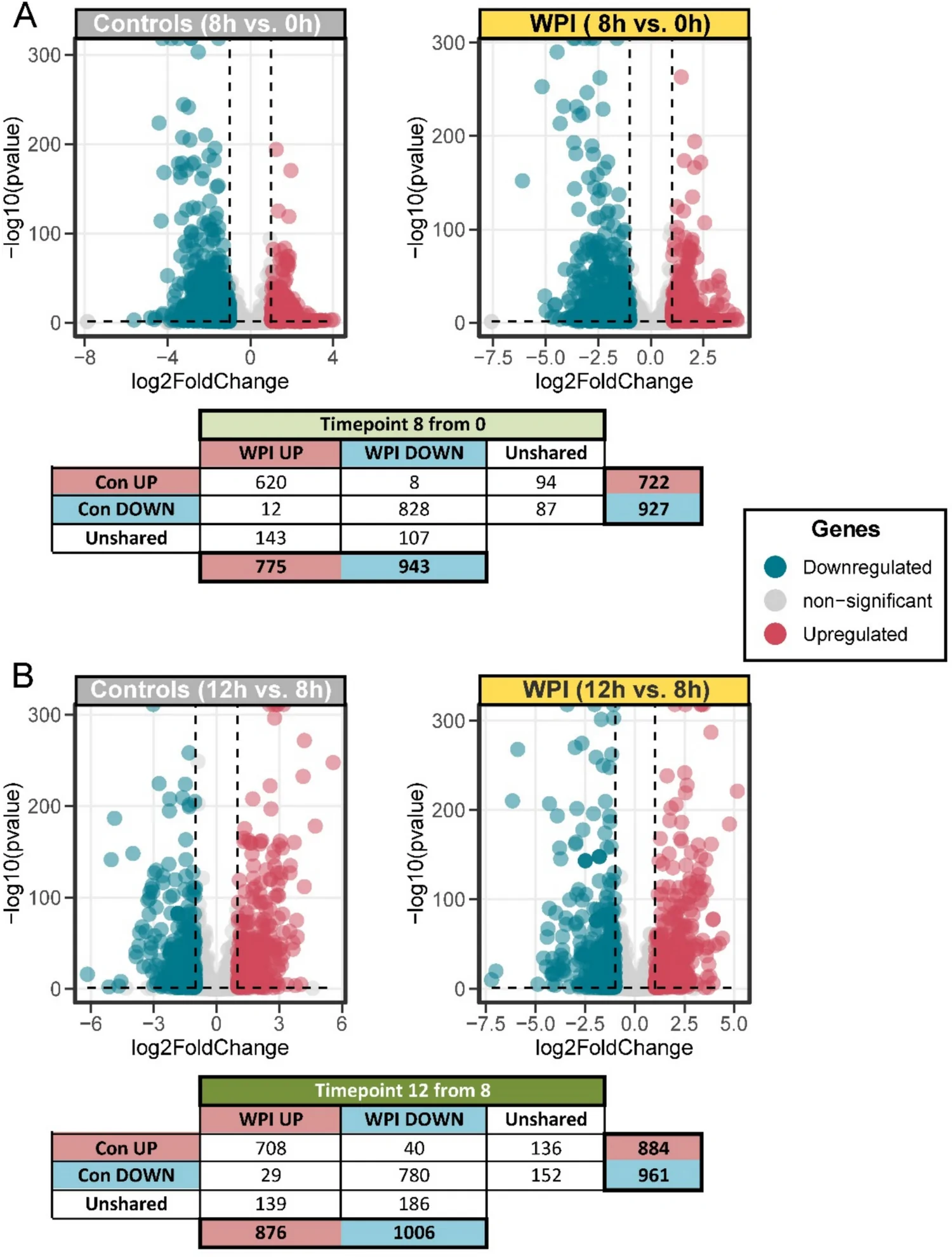
Volcano plots showing differentially transcribed genes (up or down) within treatment groups; **A** from 0 to 8-h incubation; **B** from 8- to 12-h incubation

To determine the impacts of WPI on LGG, we examined gene transcription using RNA-Seq. This analysis used the 0-, 8-, and 12-h timepoints to capture the LGG during exponential and early stationary growth. Based on normalized transcript levels, most genes were transcribed at a consistent level across the experiment, and clustering of samples by gene transcription levels produced clusters defined by timepoint rather than treatment (Fig. 2A and B, File S2). Looking more closely at the shifts in transcription with growth, at the first transition from 0 to 8 h of growth there were more downregulated genes than upregulated (Fig. 3A) in both the control samples and the WPI-amended samples, and in both cases, the majority of genes up- or downregulated were the same regardless of treatment (*n* = 620 shared upregulated genes, *n* = 828 shared downregulated genes). In the transition from the 8-h timepoint to the 12-h timepoint, there were again more downregulated genes than upregulated genes, and here too most were common to both treatment classes (*n* = 708 shared upregulated genes, *n* = 780 shared downregulated genes) (Fig. 3A). The LGG genome as analyzed here includes 2883 coding sequences, so the differentially expressed genes represented ~25–35% of the coding sequences at any time point. This underscored how dynamic transcription was during these growth phases.

**Fig. 4:**
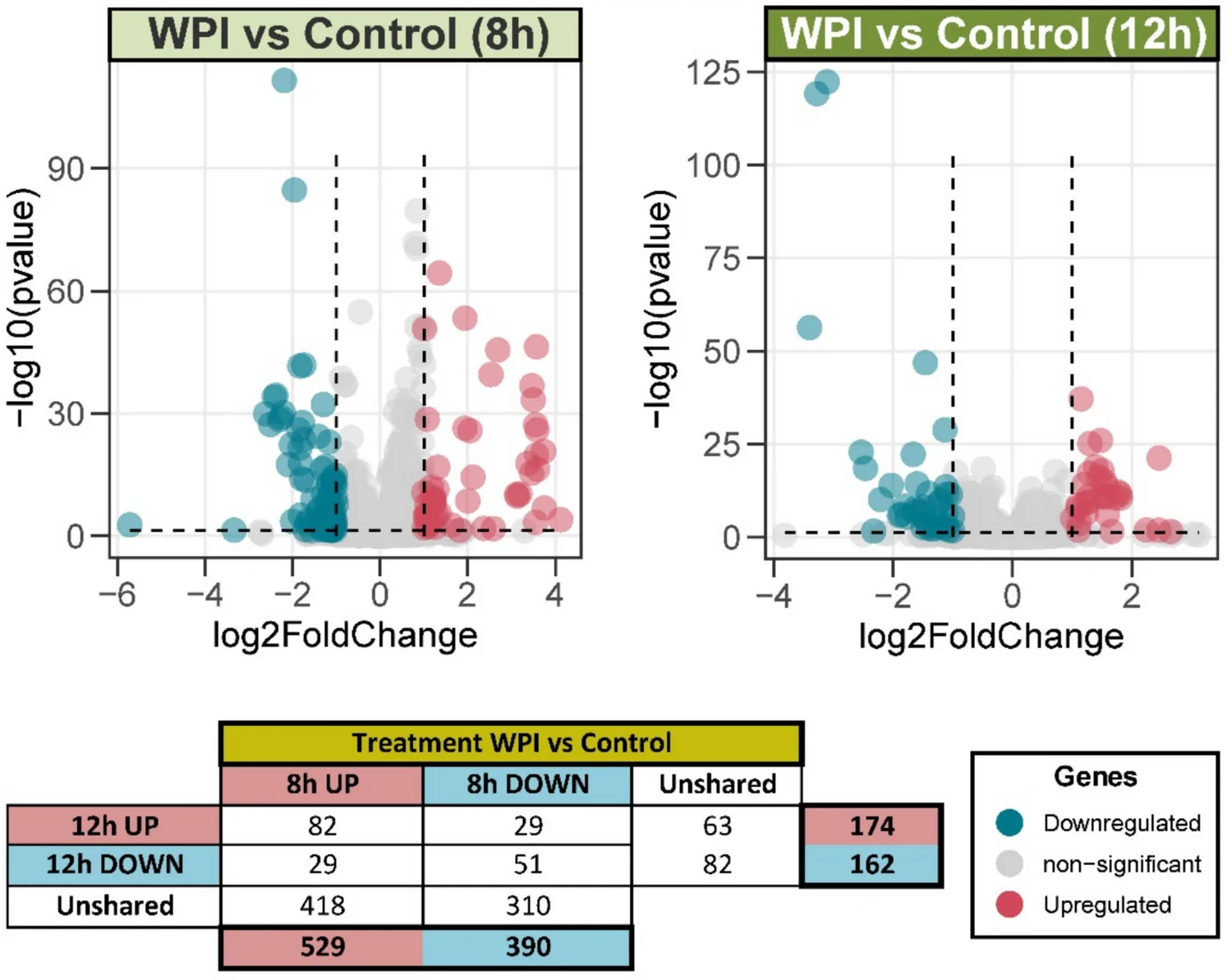
Volcano plots showing differentially transcribed genes (up or down) by treatment within the two treated timepoints. Numbers of genes shared up- or downregulated are in schematic below

Because of the strong differences in gene transcription based on timepoint, we considered it less likely that genes would be globally up- or downregulated based on WPI amendment. So, we next looked at treatment-associated transcriptional shifts separately within each of the 8- and 12-h timepoints. Overall, the number of significant treatment-responsive genes (*n* = 1064 unique genes) was lower than growth-phase responsive genes (*n* = 2500 unique genes) (Fig. 4). However, considering treatment as opposed to growth phase, there were more significantly upregulated genes than downregulated genes with counts of 529 and 174 genes upregulated, and 390 and 162 genes downregulated by WPI amendment at 8 and 12 h, respectively (Fig. 4) And as with the timepoints, the significantly different transcriptional response to WPI was much greater (2.74-fold) at the early, 8-h timepoint than later in the growth experiment. These global patterns of temporal and treatment transcriptional changes agree with the growth curves showing an increase in cell numbers by 8 h, which was further differentiated by treatment.

### Transcriptional response of gene groups of interest

#### WPI nutrient utilization

**Fig. 5:**
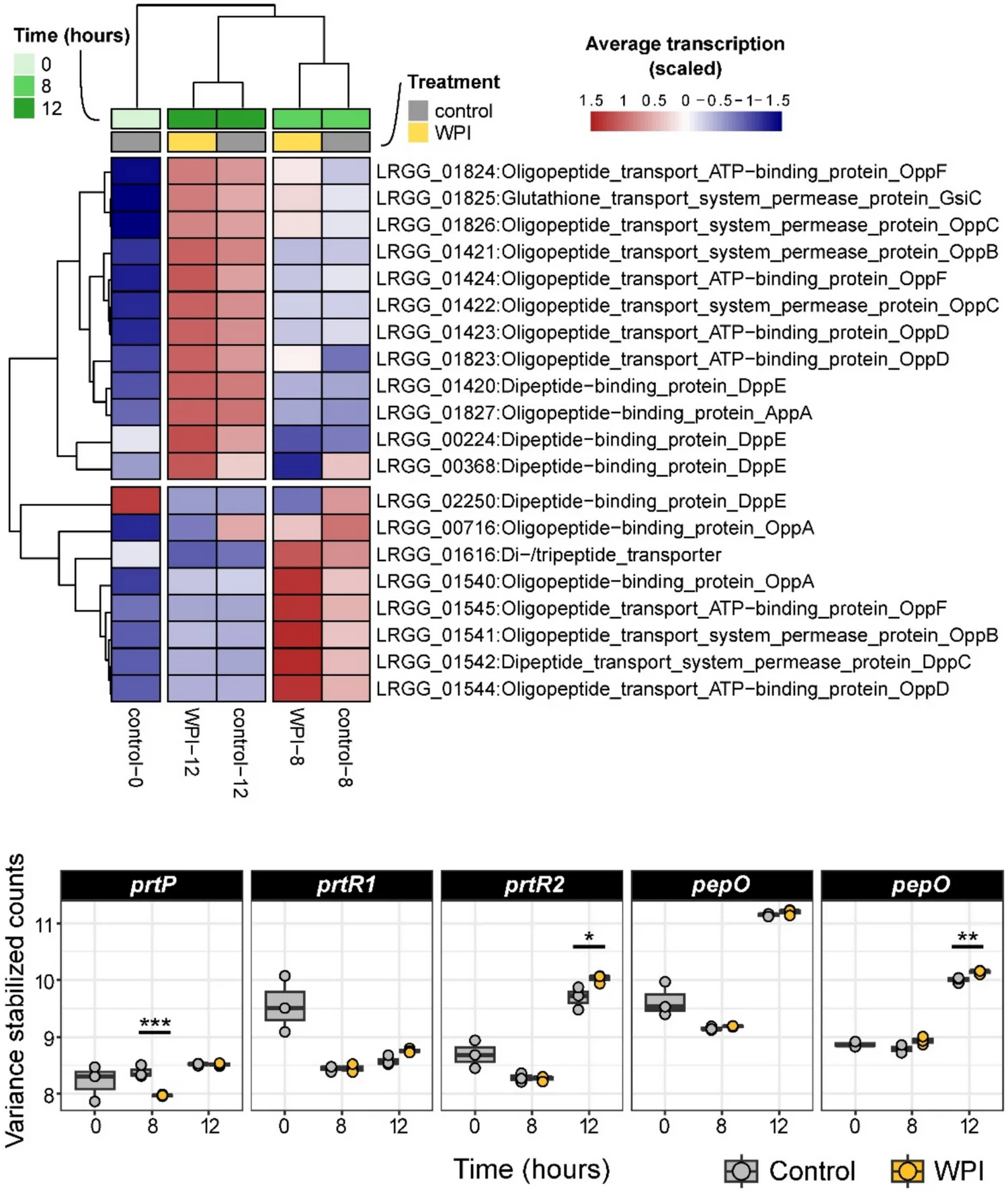
**A** Heatmap showing transcription of oligo-, di-, and tripeptide transport genes. **B** Transcription of the genes in the proteolytic system of LGG. * < 0.05; ** < 0.01; *** < 0.001. There are two *pepO* genes present in the genome

To examine the specific transcriptional response to the protein in the WPI amendment, we looked at the transcription of genes associated with peptide and amino acid import and utilization. LGG contains three *Opp* operons located approximately 120 and 280 genes apart. Most genes (18 of 20) involved in peptide transport were significantly upregulated with WPI addition at either the 8- or 12-h timepoints but only five were significantly upregulated at both, indicating that these gene sets may be regulated in either a time-dependent manner related to growth phase or to changing external levels of peptides (Fig. 5A, File S2). Peptidases displayed a similar pattern wherein most were increased at either 8 or 12 h rather than displaying a consistent pattern of transcription over time [Supplementary Fig. 3, File S2]. The LGG proteolytic system included the *prtP, prtR1*, *prtR2*, and *pepO* genes. This system displayed a growth-phase-dependent pattern with all genes being more highly transcribed at 12 h than at 8 h, with limited significant differences associated with WPI addition where the *prtP* gene was downregulated in WPI relative to control at 8 h while at 12 h two genes (*prtR2, pepO*) were upregulated in WPI relative to control (Fig. 5B, File S2).

#### Host-beneficial genes

**Fig. 6:**
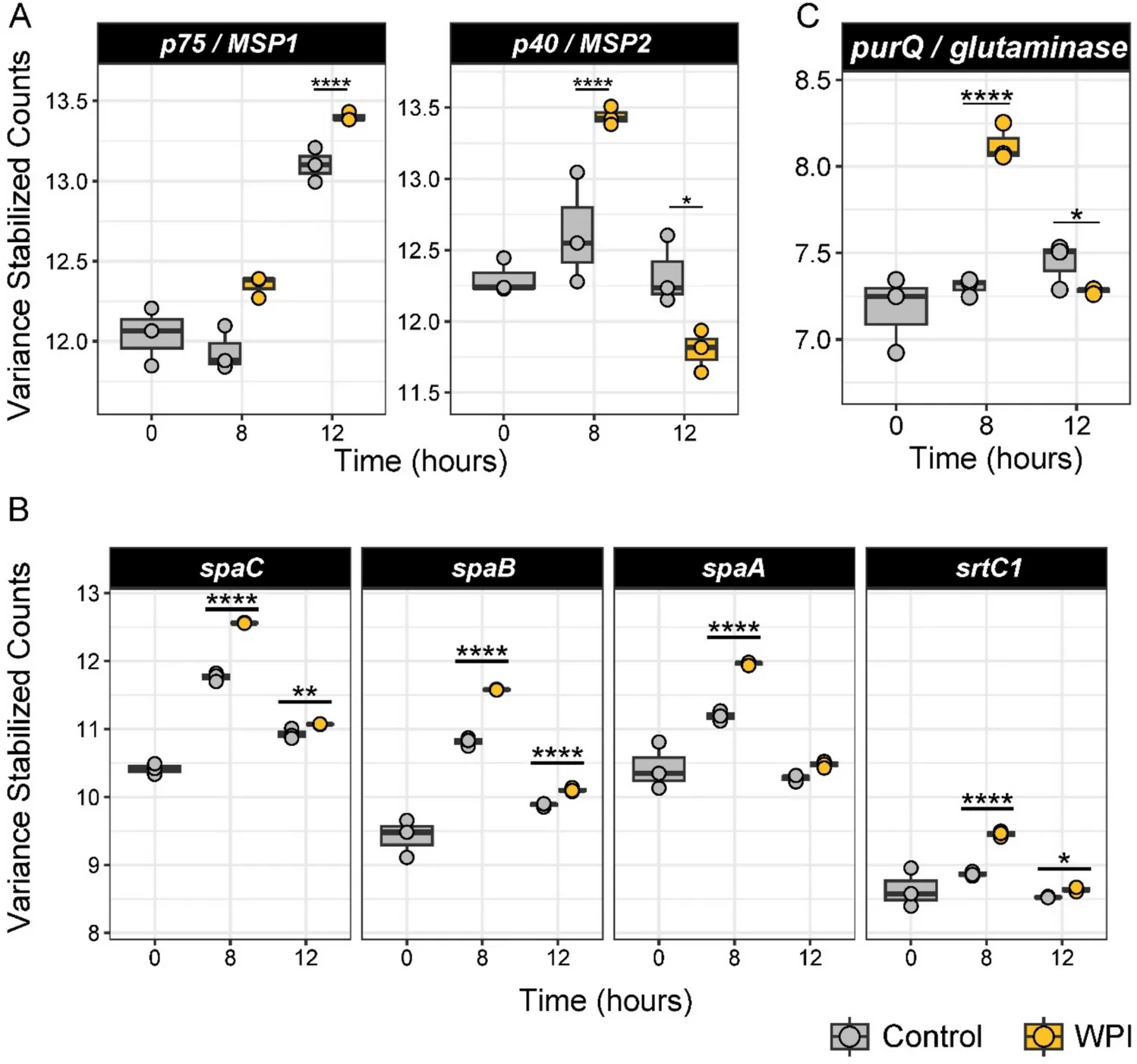
**A** Transcription of major secreted proteins MSP1 (p75) and MSP2 (p40). **B** Transcription of the adhesion pilus genes *spaCBA, srtC1*. **C** Transcription of glutaminase *purQ.* * < 0.05; ** < 0.01; *** < 0.001, **** < 0.0001

Among the proteins associated with host-beneficial effects of LGG are *MSP1* (*p75*) and *MSP2* (*p40*). Genes for these secreted proteins were among the highly differentially expressed genes with *p75* being significantly upregulated with WPI amendment at both the 8-h and 12-h timepoints relative to control samples (Fig. 6A). The *p40* gene was significantly upregulated at 8 h but was significantly downregulated at 12 h. Other host-beneficial features of LGG include an adhesion pilus which is encoded by the *spaCBA* and *srtC1* genes. In this experiment, the addition of WPI resulted in significantly increased transcription of *spaCB* and *srtC1* at both timepoints and increased transcription of *spaA* which was visible at both timepoints but achieved statistical significance only at the 8-h timepoint (Fig. 6B).

#### Other notable shifts in gene transcription

Among the most largely upregulated genes at the 8-h timepoint were those for purine metabolism. Of the 42 genes involved in purine metabolism (KEGG pathway lrh00230), 26 were significantly upregulated at the 8-h timepoint in response to WPI addition [Supplementary Fig. 4, File S2]. Of these 26 genes, 9 were later significantly downregulated at 12 h, suggesting a quick and short-lived response to the WPI addition. This set of genes includes the glutaminase *purQ* (LGG_01810) which was 10.4X more highly transcribed in WPI vs. controls at 8 h but was 0.45X lower in WPI than controls at 12 h (Fig. 6C). Like many of the genes noted above, this too suggests an immediate but short-lived response to WPI addition.

### Metabolome changes with WPI addition

**Fig. 7:**
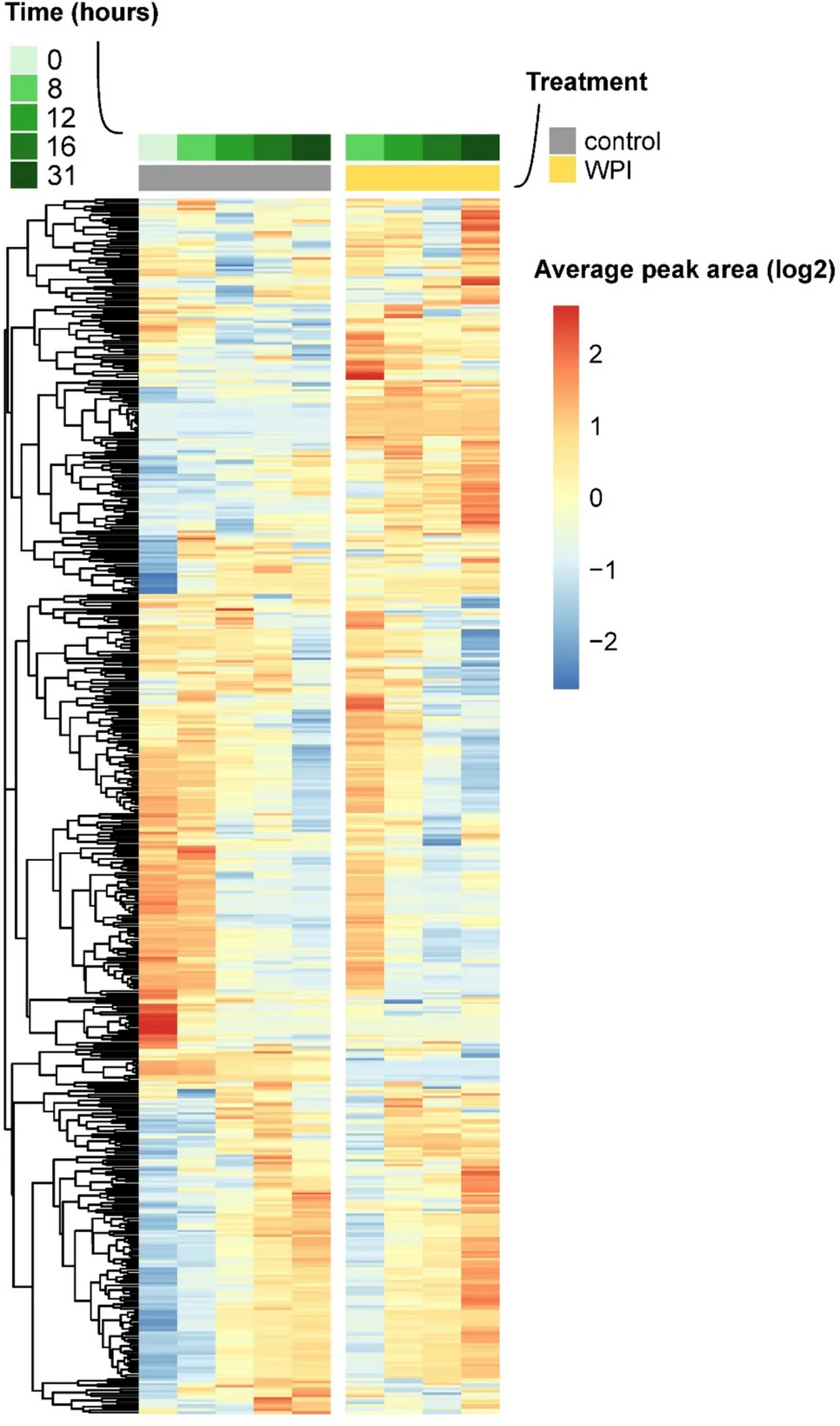
Heatmap of all identified metabolites

**Fig. 8:**
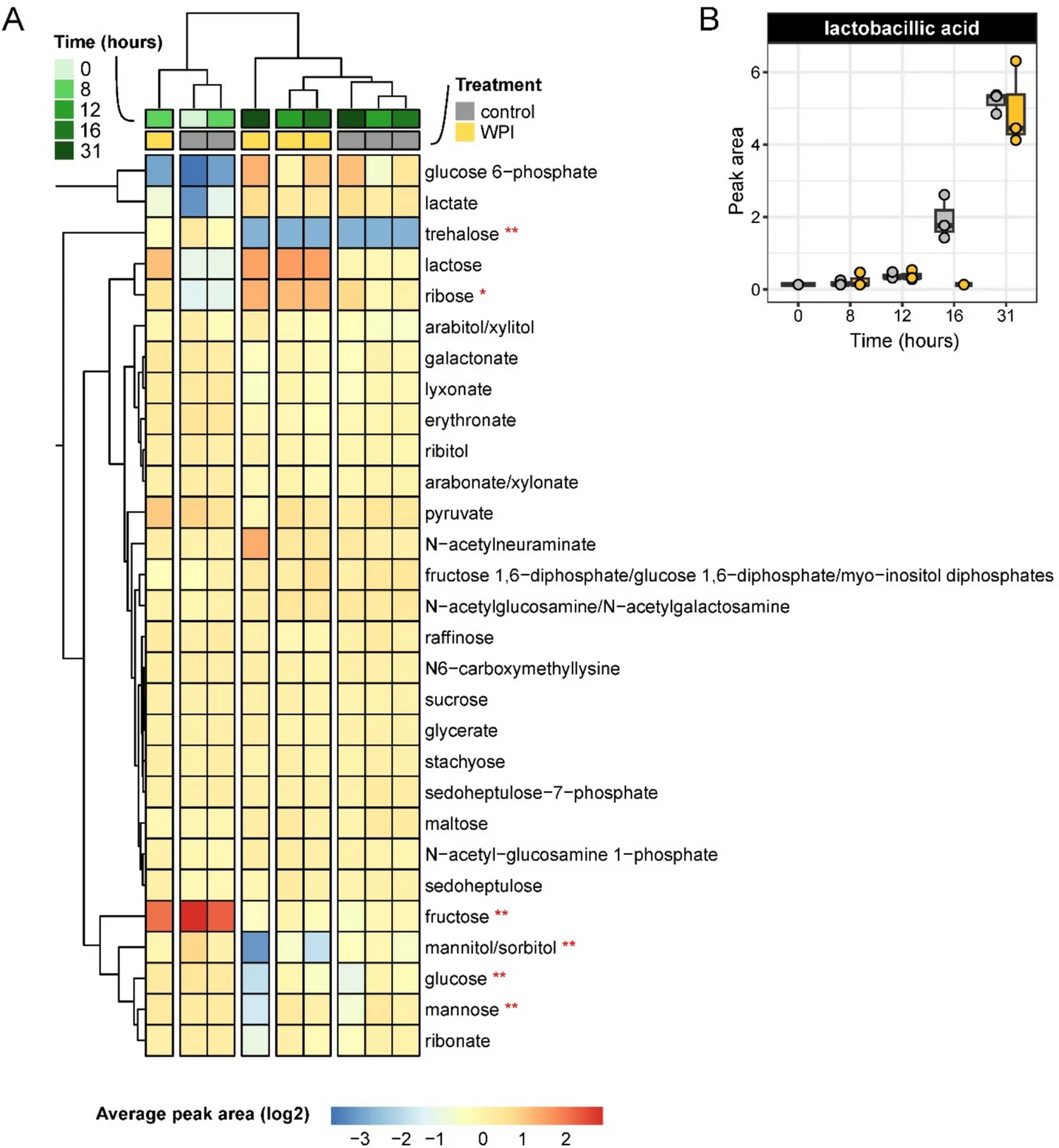
**A** Heatmap of “Carbohydrate” metabolites detected in the untargeted metabolomics data. Red asterisks indicate sugars which can be used by LGG as summarized from Douillard et al. (2013), Gorbach et al. (2017), and Arnold et al. (2018). * variable reports of fermentation, ** fermentation consistently reported. **B** Peak area of lactobacillic acid as detected in this study

The metabolite profile differed from the transcriptional profile. Here, the samples were grouped more by treatment than by time (Fig. 7). Of the 517 named metabolites, 305 differed significantly by treatment at any of the 4 timepoints [File S3]. However, only 167 of these differed at more than one timepoint (*n* = 57 at 4 timepoints, *n* = 38 at 3 timepoints, *n* = 72 at 2 timepoints.) As in the case of the genes, this signified that most metabolites which differed significantly by treatment only did so at one timepoint [File S3]. While the WPI input was not profiled, certain of the observed increased metabolites can be inferred to have derived from the WPI and were not consumed or produced by LGG. For example, 57 metabolites differed significantly at all 4 timepoints, with 49 of these belonging to the super group classifications of amino acids (*n* = 11), lipids (*n* = 14), or nucleotides (*n* = 24.) Of the 57 metabolites, several were present exclusively in the WPI samples, indicating that these were components of the WPI which were not consumed by the LGG. These included several phosphatidylcholine and phosphatidylethanolamine compounds and fatty acids [File S3]. In addition to those lipids, lactose displayed this pattern (Fig. 8A). While WPI is processed to remove sugars, some residual lactose may be present, as was seen in this experiment. Other sugars displayed apparent consumption patterns (Fig. 8A). Trehalose was minimally identified in 0- and 8-h samples but was fully consumed by timepoint 12 in both treatments. Ribose had a similar pattern to lactose and may have derived from the WPI. Fructose was identified in the early samples and diminished by hour 12. Expected metabolic outputs of LGG include lactate, which is the product of glucose or mannose fermentation. In this experiment, glucose and mannose utilization were demonstrated by the decline in glucose and mannose levels over time. Glucose levels were lower in control samples vs. WPI samples, with this difference being statistically significant in the last two timepoints, suggesting greater consumption. Glucose concentrations were highly inversely correlated with lactate concentrations (*R*^2^ = 0.865, *p* = 0.0003). Mannose depletion appeared more variable, but this too was statistically significantly lower in WPI samples at the last timepoint [Supplementary Fig. 5, File S3]. Lactate steadily accumulated over the course of the 31-h experiment, slightly but not significantly amplified by WPI addition apart from at the 8-h timepoint [Supplementary Fig. 5]. Lactobacillic acid production, a marker of entry to stationary phase, was significantly increased in the control cultures at 16 h as compared to the WPI (*p* = 0.03). At 31 h, the control samples were still higher, though the variability among replicates rendered this not statistically significant (Fig. 8B).

**Fig. 9:**
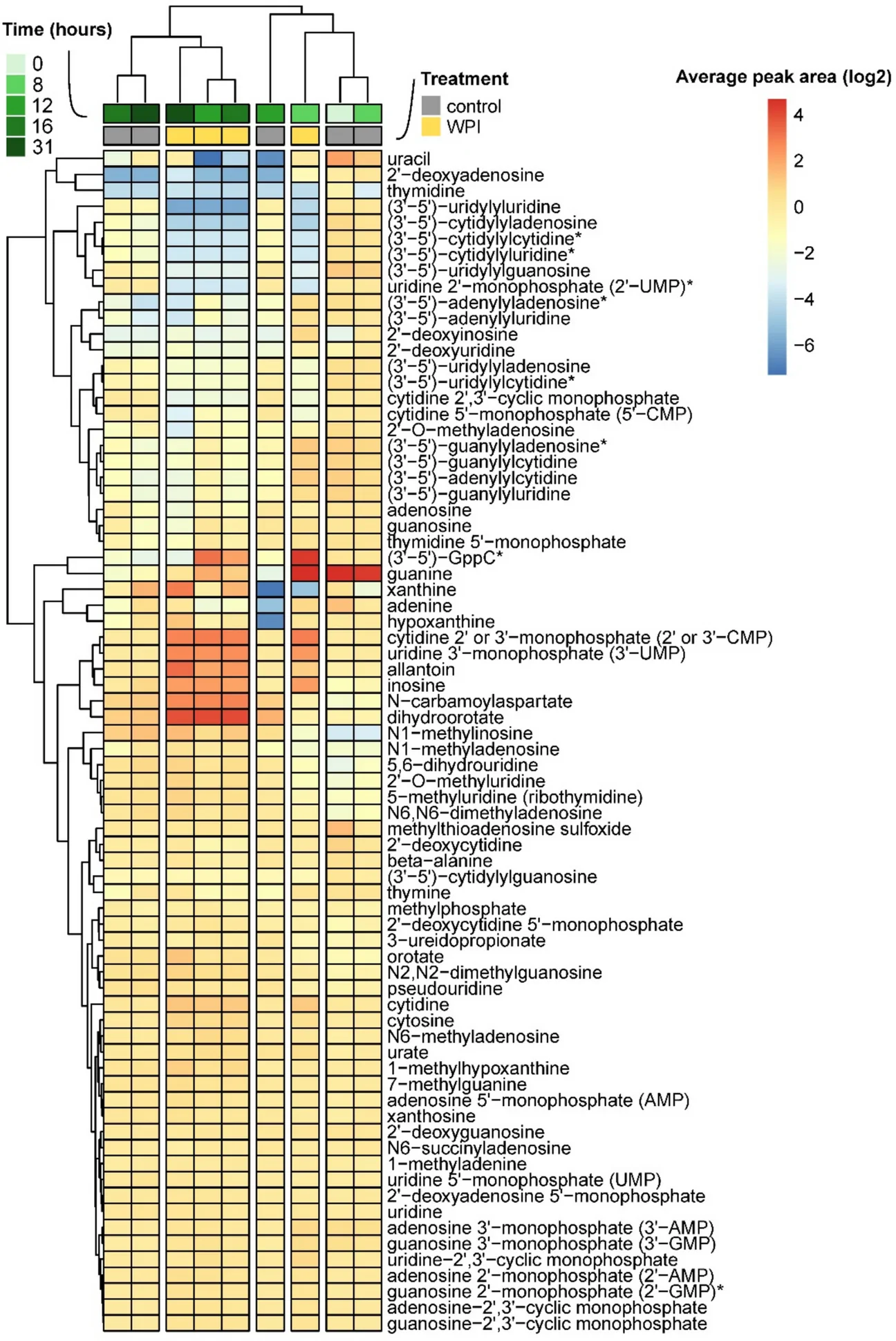
Heatmap of “Nucleotide” metabolites detected in the untargeted metabolomics data

Among other metabolites that increased with the addition of whey were purine pathway metabolites including dihydroorotate and N-carbamoylaspartate which corresponded to initial increases in transcription of genes associated with this pathway at 8 h, followed by decreases at 12 h (Fig. 9, File S3). Related to this metabolic pathway, glutamylated amino acids, glutamine, glutamate, and N-methyl GABA significantly increased in the WPI-amended samples particularly at the later timepoints, though for several of these metabolites there was a dip in accumulation at the 16-h timepoint where the WPI samples were surpassed by the control samples [Supplementary Fig. 6]. Many of the metabolites which additionally changed significantly in response to WPI addition are classified as “Peptide.” Of the 64 identified metabolites so classified, 59% (*n* = 38) differed significantly with WPI addition for at least one of the four timepoints. From these 38, 12 (*n* = 32%) contain branched chain amino acid residues [Supplementary Fig. 7].

## Discussion

In this study, we tested the effects of whey protein isolate on the growth and transcription of host-beneficial effects of *Lacticaseibacillus rhamnosus* GG. WPI is a broadly used and readily available nutritional supplement, and LGG is a widely used probiotic. Prior work considered the growth of LGG in a whey-based medium, using gene microarrays and proteomics as compared to the transcriptomics and metabolomics used here. Additionally, this previous study characterized the growth-stage shifts in LGG activity and metabolism in the whey-based medium rather than a treatment vs. control comparison (Laakso et al. 2011). Aligning with those previous results, we identified that growth-phase-related metabolic shifts are extensive, with the additional finding here that many of these effects were amplified by or only occurred due to WPI addition. These whey-sensitive features included several important probiotic aspects of LGG. These effects were observed following a single administration of 1% WPI and may not reflect effects of higher or lower concentrations.

WPI was effective at extending and enhancing the growth phase of LGG. The addition of WPI to the MRS growth medium enhanced the growth of LGG, yielding higher cell counts in the amended samples than in the controls and a moderately higher growth rate. In addition to the growth amplification of LGG by WPI, the WPI may have delayed the start of stationary phase in these cultures. The timing of the appearance of lactobacillic acid in the metabolomics data may hint to this effect. Lactobacillic acid appeared at 16 h in the control samples and appeared in the WPI samples only at the 31-h timepoint. Lactobacillic acid is a cyclopropane fatty acid the production of which can be associated with the transition from exponential to stationary phase, or with starvation conditions (Grogan and Cronan 1997). Transcription of the cyclopropane-fatty-acyl-phospholipid synthase gene (LGG_02109) did not differ significantly with treatment; however, based on the lactobacillic acid profile, this would likely have occurred at later timepoints for which RNA-Seq data was not acquired.

The enhanced growth of LGG with the addition of WPI is likely the result of additional nutrients allowing the LGG to grow beyond the depletion of nutrients in the MRS background medium. The enhanced growth of LGG in WPI medium as compared to controls was concordant with evidence of fermentation of glucose from the medium and the accumulation of lactate as the end-product. This is expected of growth of LGG in glucose-rich MRS (Bove et al. 2011). A previous study which considered the use of WPI as a coating agent for LGG found increased growth but decreased metabolic activity (Lu et al. 2021). This contrasts with our results and highlights that the method of LGG exposure to WPI and the other growth conditions will have important implications for its effectiveness. WPI is defined as > 90% protein by weight, with sugars and fats removed. This study suggested the presence of some residual lactose; however, this is not utilized by LGG and persisted in the medium. While many lactobacilli do ferment lactose, *L. rhamnosus* does not, despite containing a residual lactate dehydrogenase operon (Arnold et al. 2018; Hussain et al. 2021), so any residual lactose in the WPI would not be responsible for the WPI-induced growth in this experiment. In contrast to the lactose-fermenting lactobacilli, *L. rhamnosus* prefers fucose, likely indicative of its habitat transition to the gut environment, which is low in lactose (compared to environmental lactobacilli), but is rich in fucosylated mucins (Douillard et al. 2013). LGG is a facultatively heterofermentative strain that additionally utilizes glucose, fructose, mannose, galactose, and others (Douillard et al. 2013; Ibrahim 2016), but is unable to use rhamnose despite its name (Kankainen et al. 2009; Douillard et al. 2013; Minervini and Calasso 2022). The presence of residual lactose in the WPI leaves open the possibility that some additional sugars may have been contributed by the WPI, for example, metabolomics analysis suggests a similar pattern for ribose as lactose. The literature disagrees as to the ability of LGG to ferment ribose (Kankainen et al. 2009; Douillard et al. 2013), and here it appears that the ribose was not fermented by LGG, so its persistence (non-consumption) throughout the experiment suggests it is more likely that the enhanced growth of LGG was due to other components of the WPI, and not due to any residual sugars.

It is more likely that supplementation of the MRS medium with WPI contributed to the increase in LGG growth by the provision of additional peptides rather than the addition of fermentable carbohydrates. Lactobacilli are characterized by multiple amino acid auxotrophies and proteolytic systems which help to acquire those needed amino acids (Raveschot et al. 2018). A prior study in mice which found that gut lactobacilli counts increased in the presence of whey protein attributed this effect specifically to the amino acids threonine and cysteine (Sprong et al. 2010). Other work (Sun et al. 2019) found that supplementation with amino acids (particularly Arg, Val, Ile, Phe, Thr, Ser, Gly, Try, Tyr, and Cys) increased LGG fermentation rate in the early growth stages (0–8 h) while inferring that proteolysis starts later once these amino acids are depleted, with similar timing of proteolytic activity observed by Laakso et al. (). Here, genes for the uptake of oligo- and dipeptides increased in the WPI-treated samples more than in the untreated controls, with different operons switching on in a time-dependent manner, consistent with observations by Laakso et al. (). This apparent response to increased peptides corresponded to an increased availability of di-, tri-, and oligopeptides which occurred in a largely time-dependent manner and may reflect both production and consumption. Many of these peptides are dipeptides which are rapidly depleted in both the control and WPI samples by the 12-h timepoint at which point certain genes of the proteolytic system were observed to increase in transcription. This combined with the profile of peptide depletion in the early timepoints could indicate an early depletion of the available amino acids followed by initiation of *prtR* transcription to generate additional needed nutrients (Olvera-Rosales et al. 2023). Thus, the additional peptides and amino acids available from the WPI likely permitted an extended fermentation and growth of this probiotic strain. If lactobacillic acid production is induced by nitrogen starvation, then the additional nitrogen sources from WPI can potentially explain the delayed appearance of this marker metabolite.

Any enhanced growth of LGG due to WPI addition offers several potential benefits to the host. Among the important factors for the effectiveness of this probiotic is its ability to adhere to human epithelial cells in order to persist in the gut (Mahalak et al. 2022). LGG adheres to the human mucosa via pili which are encoded by the *spaCBA* and *srtC1* genes, with the SpaC subunit playing a critical role in adhesion to the epithelium (Kankainen et al. 2009; Ardita et al. 2014). In this study, as elsewhere (Laakso et al. 2011), the transcription of these pilus genes was found to be growth-stage-dependent, with higher levels of transcription at 8 h than at 12 h, which indicates that the LGG growth phase is important for its adherence to the mucosa. However, in addition to the time-dependent effects, these genes were significantly increased in transcription by the addition of WPI. This WPI-associated increase was particularly evident at 8 h but remained visible at the 12-h timepoint. This suggests that a nutrient infusion from WPI addition may be able to enhance the generation of the adhesion pili, enabling LGG to better establish itself in the colon mucosa. If this effect is unique to WPI as compared to other sources of additional nitrogen remains to be determined; however, in parallel with this experiment, we also tested the effects of soy protein isolate (SPI) on the growth of LGG. Growth in WPI outpaced growth in SPI at all timepoints and unlike WPI, the modest increases observed with SPI addition did not result in significantly larger cell counts by 8 h [Supplementary Fig. 2]. 

In addition to the adhesion pili, LGG produces two secreted low-molecular weight proteins, p40 (Msp2) and p75 (Msp1) (Yan and Polk 2002, 2012; Yan et al. 2007, 2013; Claes et al. 2012). In some species and strains of lactobacilli, these proteins may be involved in cell wall remodeling, but in LGG these have been demonstrated to exert host-beneficial effects by preventing epithelial barrier damage and providing an antiapoptotic effect with the potential to mitigate colitis symptoms (Yan et al. 2013, 2017; Segers and Lebeer 2014), with p40 (Msp2) being the more potent of the two. This effect was demonstrated in bacteria-free extracts but was enhanced by the presence of the LGG (Yan and Polk 2002). Concentrations of p40 and p75 have been shown to increase with fermentation (Laakso et al. 2011), and these fermentation effects were readily apparent in this study, with an increase over time in the transcription of genes encoding both proteins. WPI exerted an amplifying treatment effect, further elevating the transcription of the p40 and p75 genes, however at different timepoints. The effects of WPI were the most immediate on the p40, with a large (1.74X relative to control), significant increase at 8 h which reversed (0.67X relative to controls) at 12 h, in contrast to the p75 which was consistently more highly transcribed (1.35X and 1.23X) at both timepoints with the addition of WPI.

LGG is also studied for its production of bioactive peptides and metabolites (Raveschot et al. 2018; Olvera-Rosales et al. 2023; Suntornsaratoon et al. 2024). The metabolites GABA, glutamate, and glutamylated amino acids have bioactive properties and may be produced by lactobacilli (Bravo et al. 2011; Tette et al. 2022), and all of these demonstrated increased accumulation in the media at the later timepoints following the addition of WPI to the medium (which does contain glutamate). This would suggest the production or conversion and excretion of these host-active metabolites by LGG may be enhanced by the addition of WPI and in agreement with this, the genes for the interconversion of glutamate and glutamine were among those significantly differentially transcribed with the addition of WPI. While LGG does not encode the *gadAB* (glutamate decarboxylase) genes which generate GABA from glutamate or *puuABCD* (gamma-glutamylputrescine synthase) which produce GABA from putrescine, a slow increase in GABA and N-methyl-GABA accumulation in the medium may represent the effects of glutaminase (*purQ*) which can be responsible for the decarboxylation of glutamate in the absence of *gadAB* (Nanga et al. 2014; Li et al. 2020). Even if not converted to GABA by the LGG, the production of this neurotransmitter’s precursor glutamate is of note (Grove 2025). The amplification of lactobacilli purine metabolism in media containing milk, or milk components, has long been known (Koskenniemi et al. 2009), and the potential contribution of this effect to glutamine metabolism suggests that this metabolic attribute can be targeted with the use of WPI as a prebiotic. Further work considering the production of specific metabolites will benefit from metabolomic profiling of the input WPI. All the effects noted above are with a tested concentration of 1% WPI. It is possible that these and other effects may be impacted by either increased or decreased concentrations of WPI, and further experimentation on this parameter would be valuable. Additionally, in a closed system such as tested here, the WPI could have affected physical parameters such as pH which may have also impacted LGG growth and metabolism. In contrast, in a complex in vivo gut ecosystem (Liu et al. 2023), the LGG may face competition for the WPI, or its metabolism may otherwise shift, so the observed effects of WPI on LGG merit further exploration in the context of the more complex GIT microbial community.

While the digestion of WPI by the host and competition for this resource within the gut microbiome are important considerations which should be explored further, it is possible that simply consuming WPI together with LGG may yield some of the beneficial effects observed in this study offering an easy health-promoting option for the consumer to adopt.

## Supplementary Information

Below is the link to the electronic supplementary material.

ESM 1 (XLSX 11.3 KB)

ESM 2 (XLSX 1.76 MB)

ESM 3 (XLSX 150 KB)

ESM 4 (PDF 3.86 MB)

## Data Availability

Raw sequencing reads and the LGG genome assembly generated for this experiment can be found in the NCBI GenBank and Sequence Read Archive repositories under the BioProject accession PRJNA1327925. Raw and processed metabolomics data can be found in the Metabolomics Workbench repository under accession number ST004696.
