# Supplementary material for: Whey protein isolate enhances the growth and probiotic features of Lacticaseibacillus rhamnosus GG
Source: Appl Microbiol Biotechnol. 2026 Jul 9;110:295. doi: 10.1007/s00253-026-13945-4 (PMC13635073; doi:10.1007/s00253-026-13945-4)
Supplement: Supplementary file 1 [file 253_2026_13945_MOESM4_ESM.pdf]

Supplementary Materials for:

Whey protein isolate enhances the growth and probiotic features of *Lacticaseibacillus rhamnosus* GG

Adrienne B. Narrowe[*], LinShu Liu[*], Venkateswari J. Chetty[†], Karley K. Mahalak, Jenni Firrman, Johanna M.S. Lemons

Dairy and Functional Foods Research Unit, Eastern Regional Research Center, Agricultural Research Service, United States Department of Agriculture, Wyndmoor, PA, USA

† - Current address: Foreign Animal Disease Research Unit, National Bio and Agro-Defense Facility, Agricultural Research Service, United States Department of Agriculture, Manhattan, KS, USA

* - Corresponding authors:
Adrienne Narrowe adrienne.narrowe@usda.gov ; LinShu Liu linshu.liu@usda.gov

Supplementary Fig. 1 Experimental design

Supplementary Fig. 2 Effects of SPI compared to the effects of WPI

Supplementary Fig. 3 Heatmap showing the scaled transcription levels peptidase genes

Supplementary Fig. 4 Heatmap showing the scaled transcription levels of genes associated with purine metabolism.

Supplementary Fig. 5 Peak areas of glucose, mannose, and lactate from the untargeted metabolomics data

Supplementary Fig. 6 Glutamine, glutamate, and gamma-glutamyl amino acids detected in the untargeted metabolomics data

Supplementary Fig. 7 Heatmap of di- and tripeptides detected in the untargeted metabolomics data

File S1 Flow-cytometry cell counts

File S2 DESeq2 results for differential transcription testing. Table includes gene annotations and ID mappings to reference strains

File S3 Tests for significant differences in metabolite areas based on treatment within timepoints

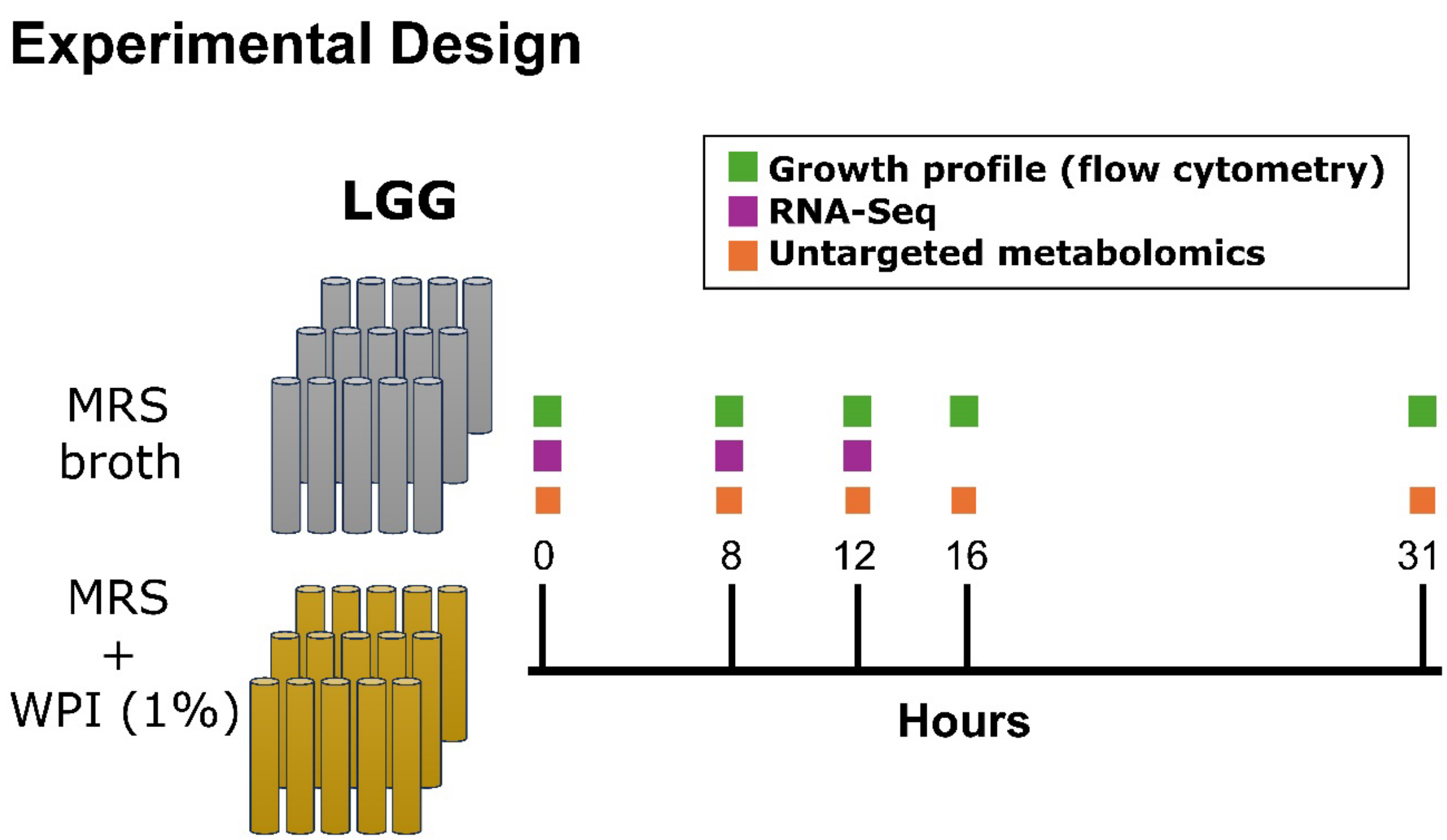

Supplementary Fig. 1 Experimental design

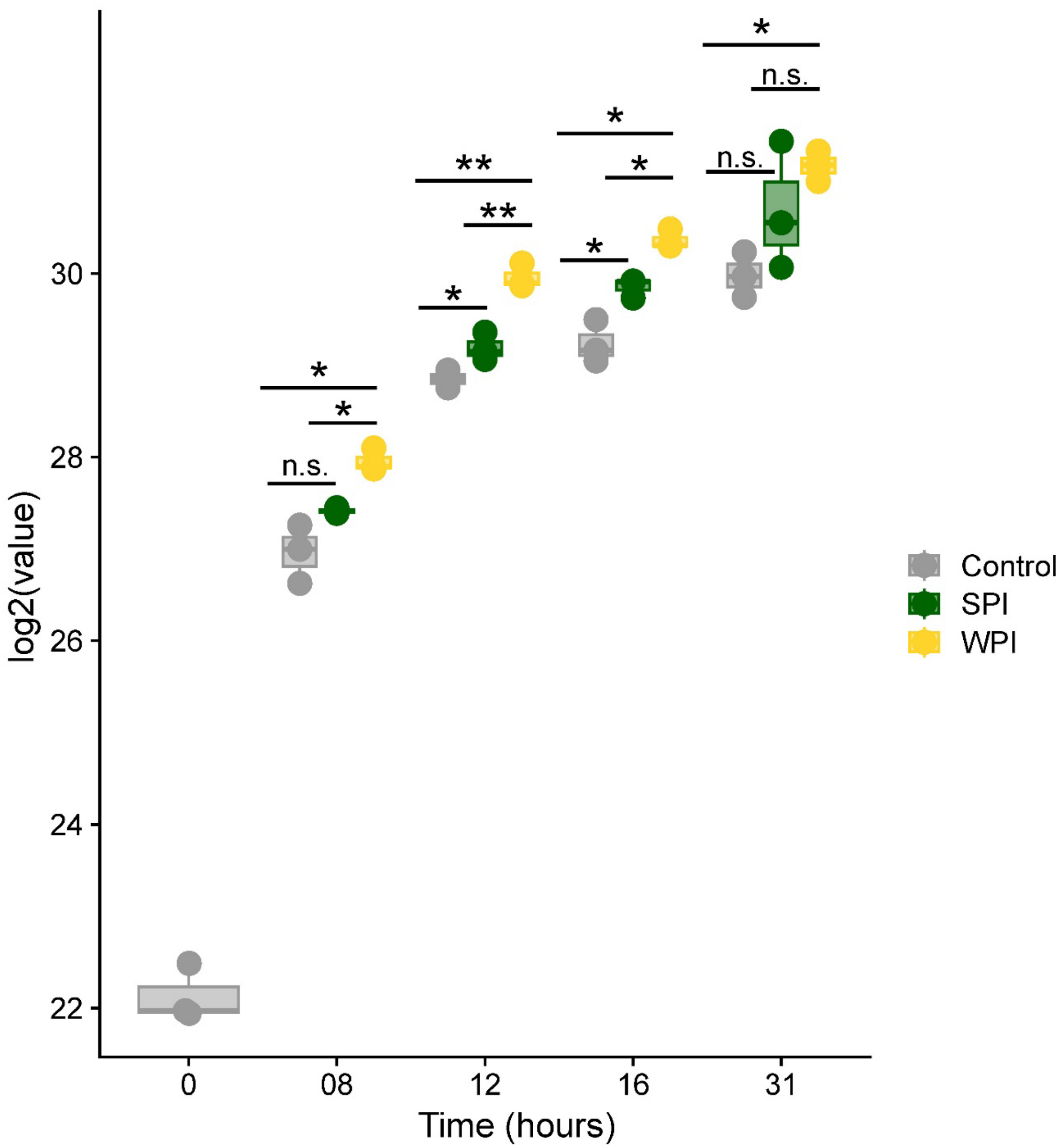

Supplementary Fig. 2 Effects of SPI compared to the effects of WPI

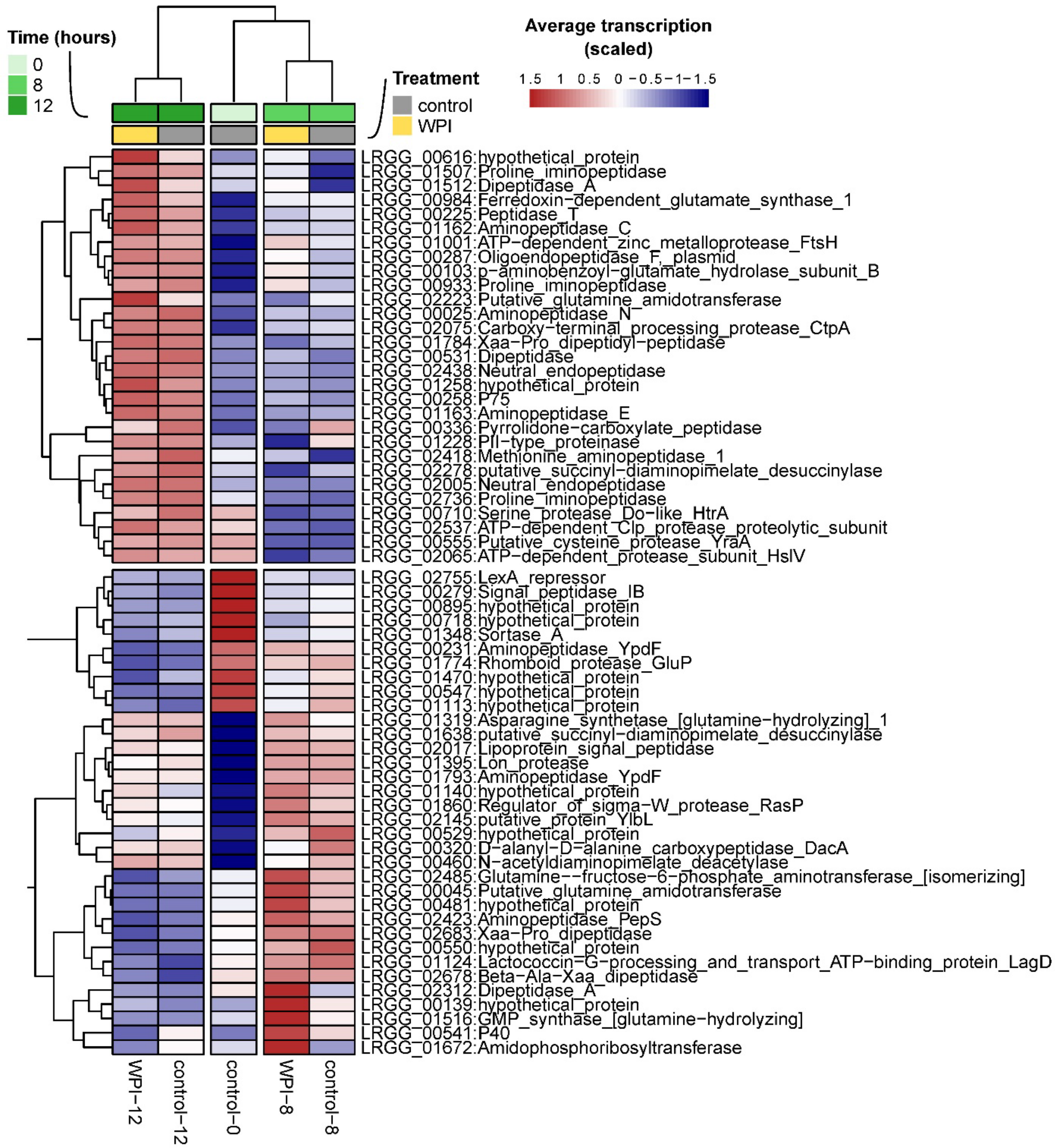

Supplementary Fig. 3 Heatmap showing the scaled transcription levels peptidase genes

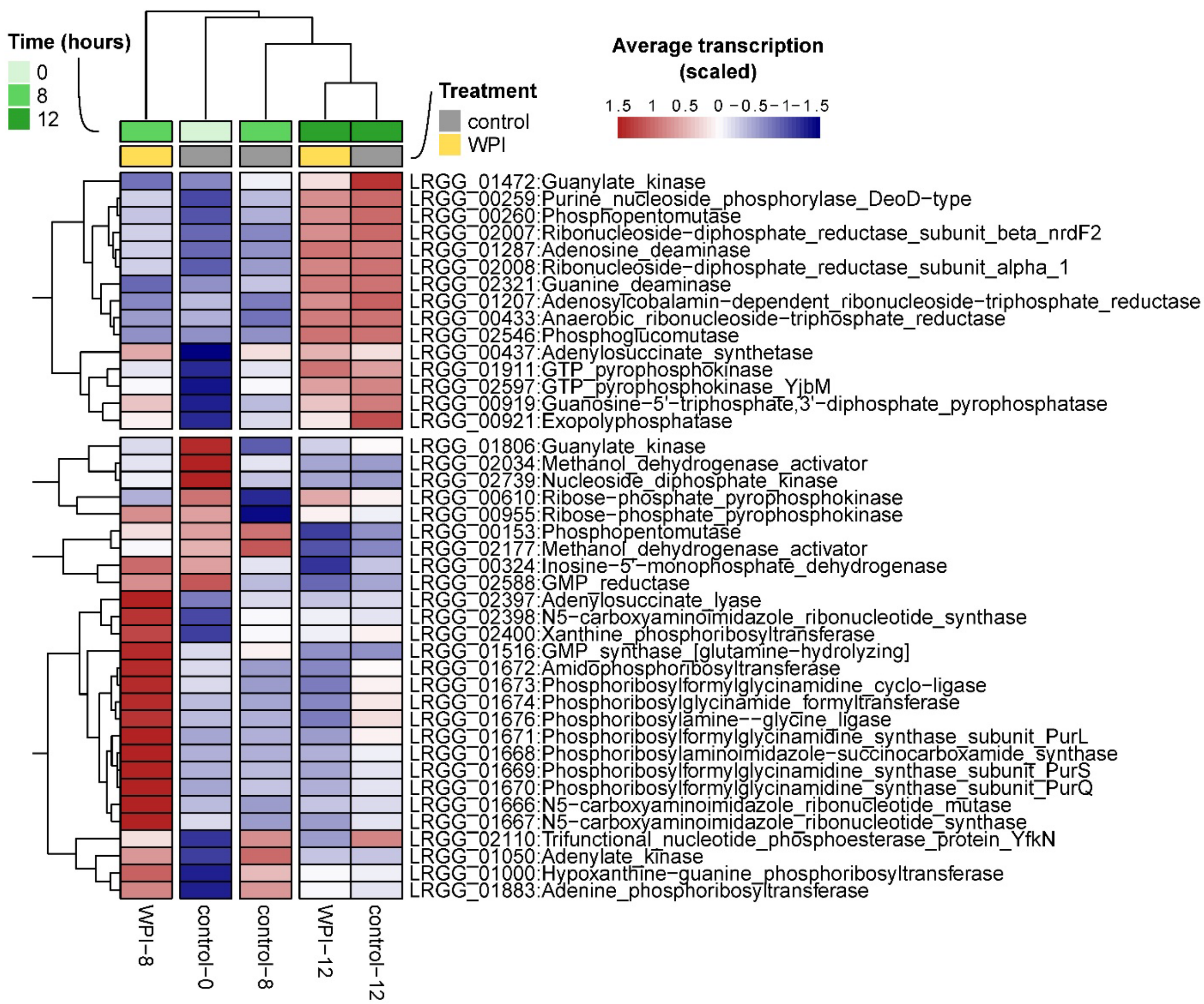

Supplementary Fig. 4 Heatmap showing the scaled transcription levels of genes associated with purine metabolism

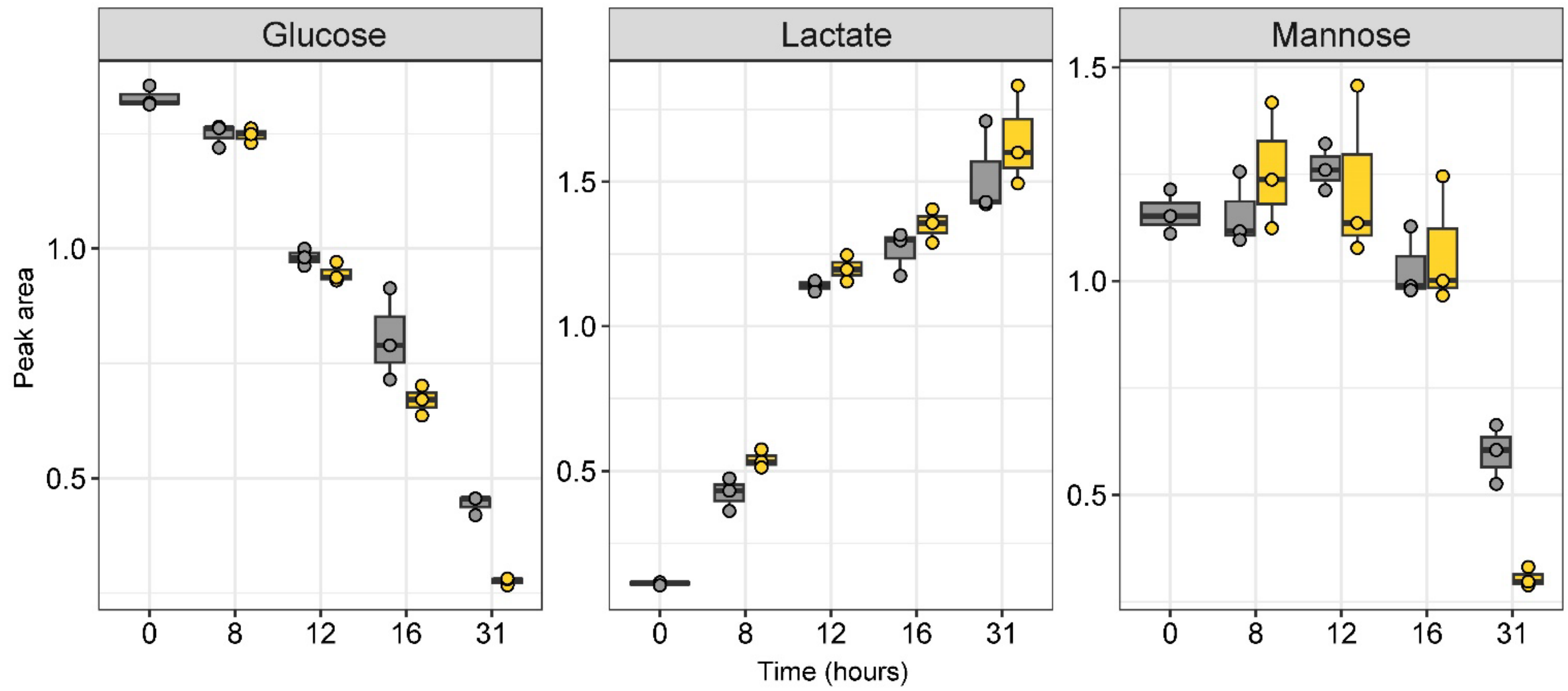

Supplementary Fig. 5 Peak areas of glucose, mannose, and lactate from the untargeted metabolomics data

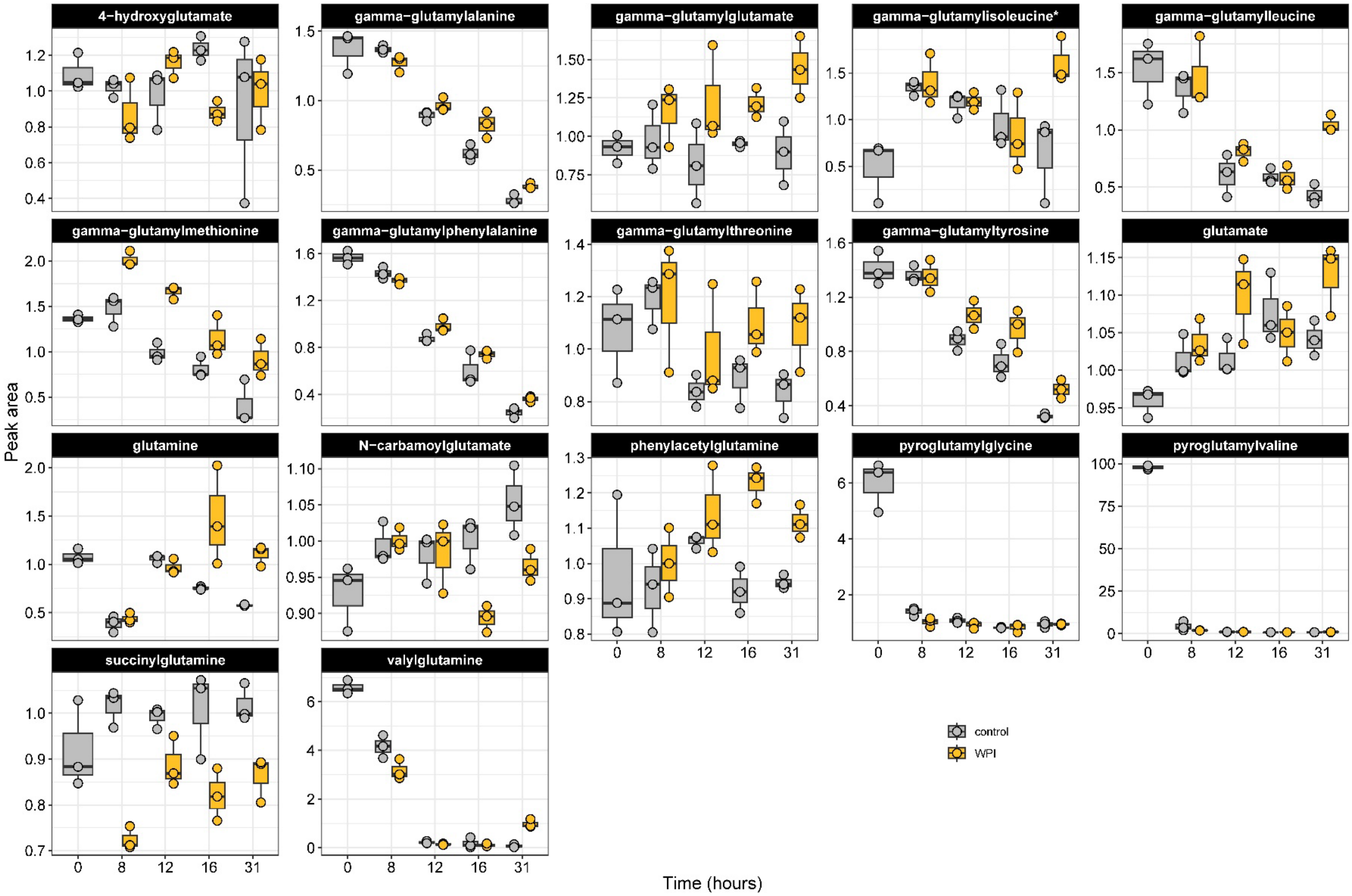

Supplementary Fig. 6 Glutamine, glutamate, and gamma-glutamyl amino acids detected in the untargeted metabolomics data

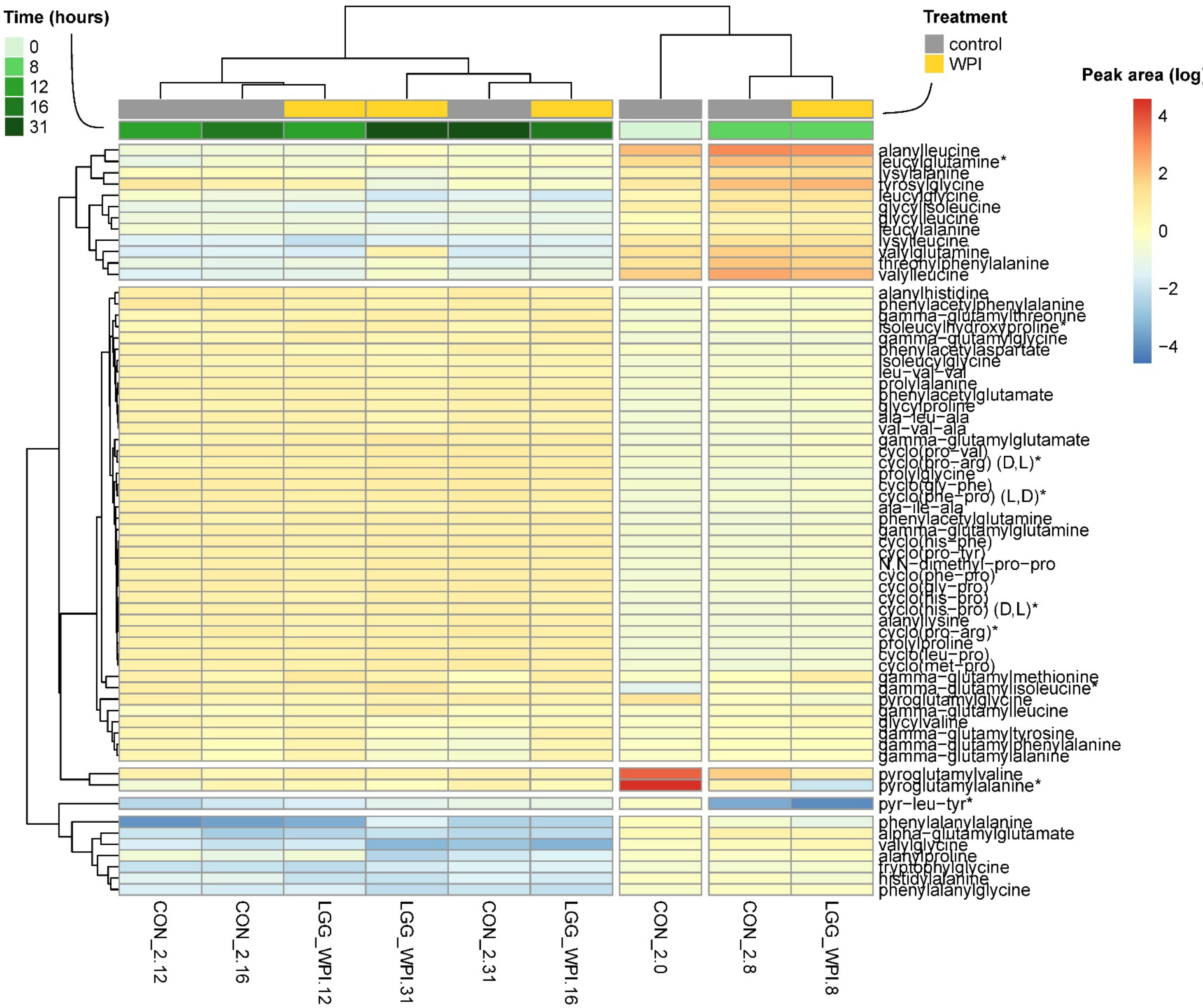

Supplementary Fig. 7 Heatmap of di- and tripeptides detected in the untargeted metabolomics data
